# Prospective evaluation of serum procalcitonin in critically ill patients with suspected sepsis- experience from a tertiary care hospital in Pakistan

**DOI:** 10.1016/j.amsu.2018.10.004

**Published:** 2018-10-05

**Authors:** Sibtain Ahmed, Imran Siddiqui, Lena Jafri, Madiha Hashmi, Aysha Habib Khan, Farooq Ghani

**Affiliations:** Department of Pathology & Laboratory Medicine, Aga Khan University, Pakistan

**Keywords:** Procalcitonin, Blood culture, Sepsis, Intensive care unit

## Abstract

**Background:**

Sepsis is the leading cause of mortality in critically ill patients. Procalcitonin (PCT) is a promising marker for identification of bacterial sepsis. The aim of this study was to determine the diagnostic accuracy of serum PCT concentration in patients with suspected sepsis admitted to mixed medical-surgical Intensive care unit (ICU).

**Material and methods:**

A cross-sectional study conducted at section of Chemical Pathology, Department of Pathology and Laboratory Medicine and ICU. Patients with suspected sepsis were included, serum PCT cut off ≥0.5 ng/ml was taken for diagnosing sepsis. Diagnostic accuracy was measured in terms of sensitivity, specificity, positive predictive value (PPV) and negative predictive value (NPV) taking blood culture as gold standard. Furthermore, different cut offs were compared by using receiver operating characteristic curves (ROC). Data analysis was done on SPSS version 20.

**Results:**

Median age of the study group (n = 103) was 48 years (IQR: 22), 60% being males. Out of the 103 patients included 82 patients had PCT levels above the optimal cut off. At a serum PCT cutoff of 0.5 μg/L, the sensitivity and specificity for the diagnosis of sepsis was found to be 93.75% and 43.59% respectively. NPV was higher compared to PPV making PCT a reliable marker to for the screening out of sepsis patients. Furthermore, it was revealed that PCT having an AUC = 0.70 outperformed WBC (AUC = 0.5) and CRP (AUC = 0.6).

**Conclusion:**

Elevated PCT concentration is a promising indicator of sepsis in newly admitted critically ill patients capable of complementing clinical signs and routine laboratory parameters.

## Highlights

• This study demonstrates Procalcitonin to be a promising marker of sepsis in critically ill patients.
• Procalcitonin was found to have better diagnostic accuracy compared to White blood cell count and C - reactive protein.
• PCT cut-off of 1.5 μg/L has better specificity for diagnosing sepsis when compared to a cut-off of 0.5 μg/L; at the cost of sensitivity.

## 1 Introduction

The past century has witnessed a rising trend in the incidence of infections, sepsis, and septic shock regardless of overwhelming development in treatment modalities [1]. Sepsis is a common condition that exists in patients admitted to intensive care units (ICU). Audits of ICU worldwide showed that 29.5% patients had sepsis on admission or during the ICU stay [2]. According to a study undertaken in 150 ICUs across 16 Asian countries; 28.3% of all admissions in participating units from Pakistan had sepsis [3].

Microbiological culture testing remains the traditional gold standard diagnostic modality for identification of sepsis with its own limitations. Prolong turnaround times for culture and the fact that blood cultures detect bacteraemia in only about 50% of patients with clinical suspicion of sepsis pose diagnostic challenge for septic patients in ICU [4]. Diagnosis is optimised by using biochemical tests for sepsis, such as C - reactive protein (CRP) or leukocyte count (WBC) which have reportedly low diagnostic accuracy and are at times ambiguous [5]. Search for new markers for providing early diagnosis with improved sensitivity and specificity continues. Procalcitonin (PCT) has developed as an ideal biomarker for sepsis and early detection of bacteraemia as it can be quantified and promptly interpreted in light of clinical context to aid decision making in patient care [6]. PCT has been used as marker of sepsis with sensitivity and specificity of 83% and 62% respectively with significantly high levels in the patients having sepsis and positive blood culture results than with culture negative results [7,8].

PCT is a glycoprotein present in C cells of thyroid gland. It belongs to the group of related peptide (C-GRP) encoded by the CALC-1 gene and is formed from the common precursor pre-calcitonin [9]. In healthy subjects, CALC-1 genes synthesize Calcitonin, but presence of microbial infection through endotoxin or pro-inflammatory cytokines increases calcitonin gene expression and PCT mRNA is mostly synthesised. This leads to release of PCT from all parenchymal tissue, exclusively in response to bacterial infection only and not viral or inflammatory disease [10]. This makes PCT to be a specific diagnostic marker to detect bacterial sepsis. On the other hand serum levels of PCT increase briskly within 2–6 h after the stimulus making it a rapid diagnostic marker compared to culture [11,12].

As the expression of PCT depends upon the genetic framework of the population, therefore, it is important to assess its role and utility in Pakistani population with different metabolic and biochemical makeup. The aim of this study was to determine the diagnostic accuracy of PCT in diagnosis of bacterial sepsis in critically ill patients admitted at ICU of Aga Khan University Hospital Karachi (AKUH-K), by taking blood culture as gold standard.

## 2 Methods

This interdisciplinary cross sectional study was conducted in the Section of Chemical Pathology, Department of Pathology & Laboratory Medicine and mixed medical-surgical ICU of AKUH-K, Pakistan. The study was approved by the institutional Ethical Review Committee (1573-Pat-ERC-11). Our study was registered with NIH clinicaltrials. gov (Unique ID: NCT03506152). The work has been reported in line with the Strengthening the Reporting of Cohort Studies in Surgery (STROCSS) criteria [13].

### 2.1 Inclusion criteria

Adult male and female patients between 18 and 70 years of age with a clinical suspicion of sepsis as guided by the ICU physicians were recruited through non-probability purposive sampling, within 24 h of ICU admission during January to December 2014. The criteria of suspected sepsis was used for labelling the cases included the presence of any two or more of the following conditions at the time of admission: temperature ≥38 °C or <36 °C, heart rate >90 beats/min, respiratory rate >20 breaths/min, white blood cell count <4 × 10^9^/L (<4000/mm³), >12 × 10^9^/L (>12,000/mm^3^). Sepsis was further confirmed by the presence of positive blood culture. Furthermore other markers of sepsis including CRP levels, WBC counts and blood culture undertaken at the time of admission as a part of routine care were also recorded.

### 2.2 Exclusion criteria

Patients above 70 years of age were excluded due to ‘immune senescence’ in elderly giving rise to different presentation of bacteraemia. Furthermore, patients discharged before 24 h, patients who had blood transfusion before ICU stay and those with organ failure were excluded from the study.

Data was collected on predesigned forms by the team of primary investigators. Patients were recruited after informed consent from immediate family members/care takers.

Seven ml of blood was drawn in gel separator tubes within 24 h of admission in ICU for PCT determination. Blood samples were centrifuged at 3000 rpm and serum was separated and stored at −20 °C until assayed. Serum PCT was measured by Electro- Chemiluminescence immunoassay (ECLIA) on the Roche Elecsys E170 immunoassay analyser using manufacturer's recommendations. Results are expressed as micro gram of PCT per litre of serum (ug/L). For internal quality control 2 levels of manufacturer provided controls [low and high] were run with each batch of analyte while samples from College of American Pathologists (CAP, USA) were run for external quality control. PCT levels >0.5 μg/L are considered positive for diagnosis of sepsis.

A sample size of 103 was calculated by using PASS 11 word home edition software by taking the sensitivity 83%, specificity 62%, Prevalence 37.4% [14] and precision 10%.

Data analysis was done on SPSS version 20. Shapiro Wilk test was used to check normality of data. As the data that was skewed; median values were reported along with interquartile ranges (IQR) for quantitative variables. Diagnostic accuracy of PCT was calculated on the basis of sensitivity, specificity, PPV and NPV taking blood culture as gold standard. For further analysis, Receiver operative characteristic (ROC) curve was plotted for PCT and area under the curve (AUC) calculated. Furthermore, association between CRP, WBC and PCT was done using ROC curve and respective AUC were compared.

## 3 Results

**Fig. 1:**
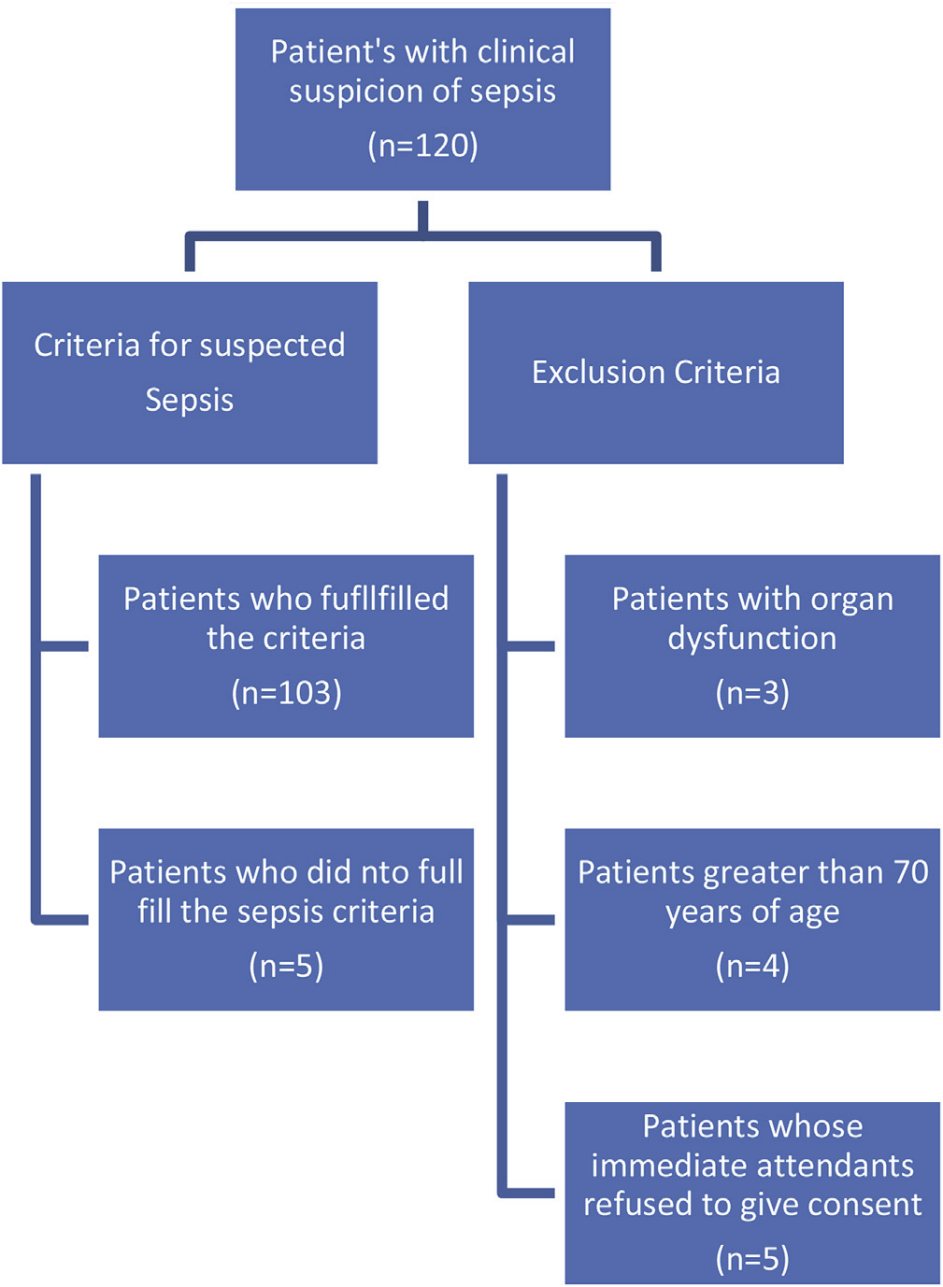
Distribution of consort showing critically ill patients included in final analysis with suspected sepsis from medical and surgical ICU.

One hundred and three patients met inclusion criteria and were included in the final study analysis as shown in Fig. 1. Sixty percent of the patients were males (n = 62). The median age of the group was 48 years (IQR: 22) and BMI was 25.4 kg/m^2^ (IQR: 4.22). Out of the 103, 43.7% were obese and 8.7% were overweight according to Asian BMI classification [15]. Median PCT levels were 1.5 μg/L (IQR: 8.5 μg/L), 79% (n = 82) patients had PCT levels >0.5 μg/L (median PCT 2.7 μg/L).

**Fig. 2:**
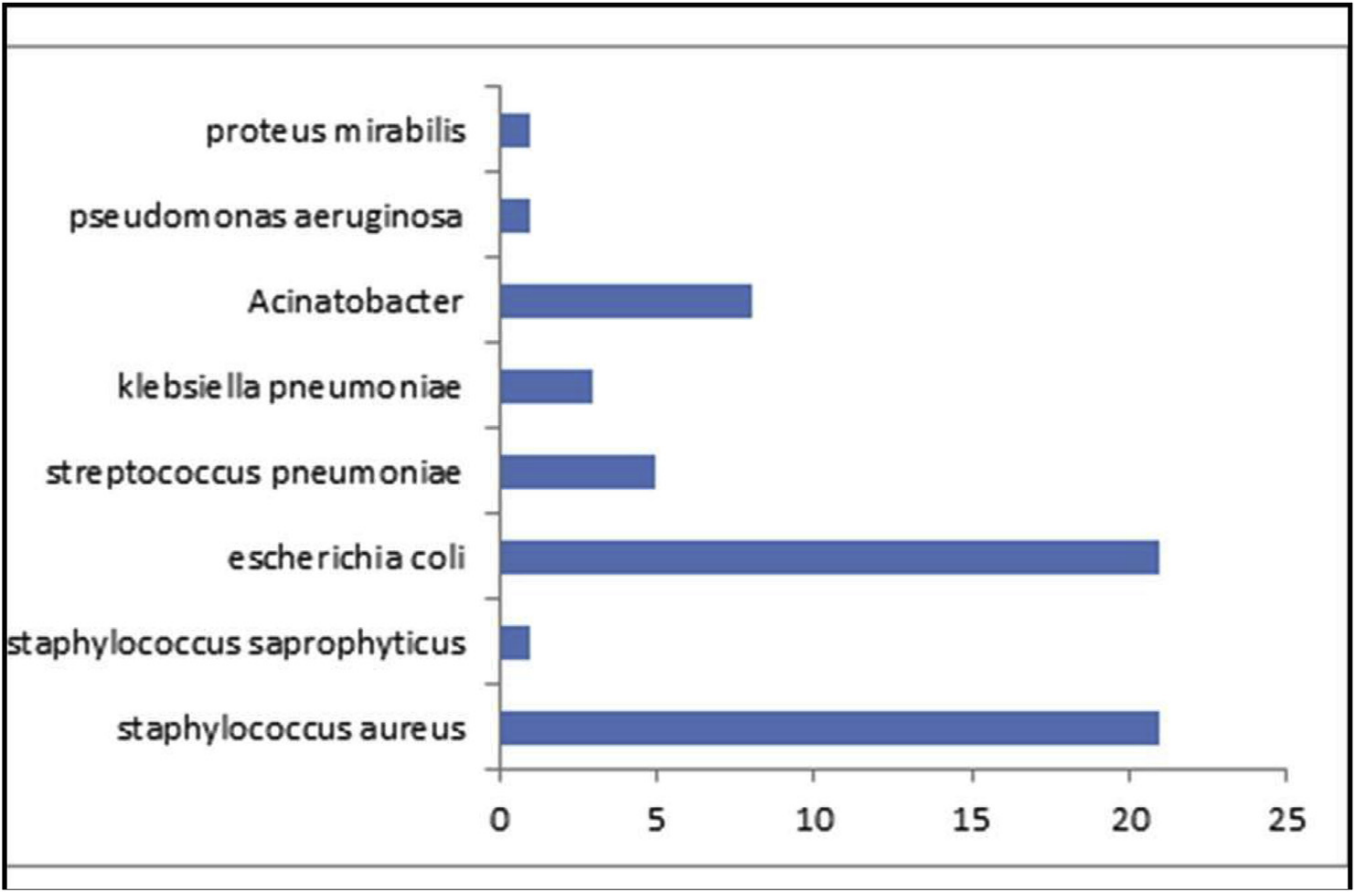
Showing culture results (n = 64).

Sixty four patients had bacterial growth on blood culture while 39 samples did not show growth over a period of 5 days. Among the various organisms identified on culture *Escherichia coli* (E.Coli) (n = 21) and Staphylococcal infections (n = 22) were the most frequent as depicted in Fig. 2.

PCT levels with a median value of 2.14 μg/L in the culture positive group, were found to be significantly higher compared to culture negative group (p value < 0.01). Furthermore, median CRP levels and median WBC levels were also higher in the culture positive groups as shown in Table 1. The sensitivity and specificity for the diagnosis of sepsis compared to culture as gold standard was found to be 93.75% (95% CI: 84.76%–98.27%) and 43.59% (95% CI: 27.81%–60.38%) respectively. NPV was calculated to be 80.95% and was higher compared to PPV (73%) making PCT a better marker for screening of patients with sepsis.

**Table 1:** Comparison of Biomarkers of Sepsis in Culture Positive and Negative Groups in critically ill patients Admitted in ICU [n = 103].

Biomarker [Reference Range]	Culture positive group [n = 64]	Culture negative group [n = 39]	p- value
	Median [IQR]	Median [IQR]	
**PCT** [< 0.5 μg/L]	2.14 [10.21]	0.8 [3.76]	0.001^a^
**CRP** [0–5 mg/L]	98 [125]	86 [138]	0.239
**WBC** [4.0-10 × 10^9^/L]	14.3 [10.53]	12.8 [8.2]	0.314

a p value < 0.01 = highly significant.

**Fig. 3:**
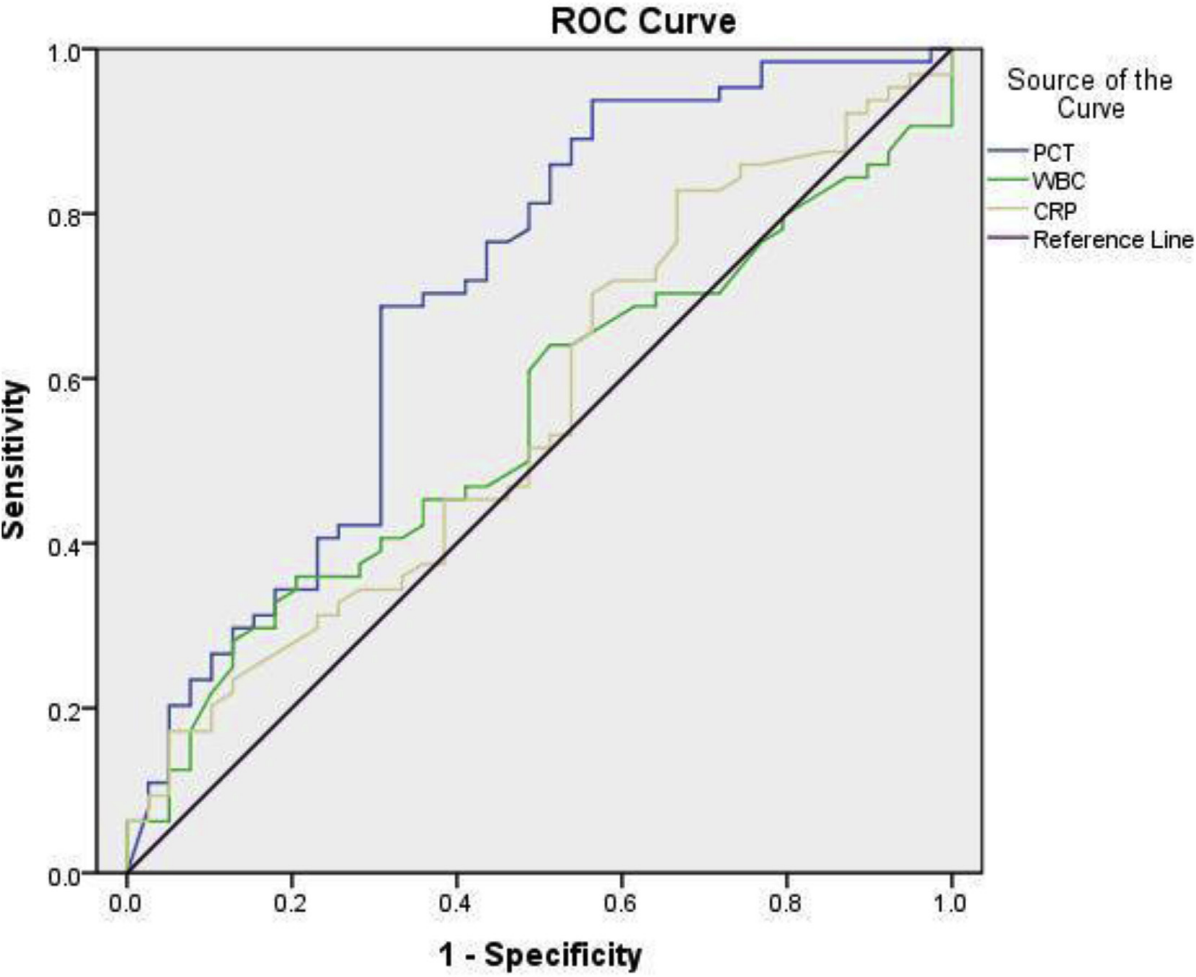
ROC analysis of PCT, CRP and WBC against culture as gold standard. (For interpretation of the references to colour in this figure legend, the reader is referred to the Web version of this article.)

ROC curve analysis was used to compare the performance of PCT with the other two markers of sepsis recorded for each study participant. PCT having an AUC = 0.7 outperformed WBC (AUC = 0.5) and CRP (AUC = 0.6). Fig. 3 illustrates the ROC curve results for PCT, WBC and CRP compared to culture results.

Diagnostic performance of PCT at various cut offs was evaluated. Performance of PCT at various cut offs is shown in Table 2. PCT cut-off of 1.5 μg/L has better specificity for diagnosing sepsis when compared to a cut-off of 0.5 μg/L; at the cost of sensitivity.

**Table 2:** Performance of PCT at various cut offs for sepsis diagnosis compared to culture.

PCT Cut off [μg/L]	Sensitivity [%]	Specificity [%]
0.5	93.8	43.5
1	71.9	57
1.5	64	70
2	51	70
2.5	51	70

## 4 Discussion

To date no gold standard test has been identified for diagnosis of sepsis because culture results may be affected by various pre analytical issues including pre-treatment with antibiotics and inadequate and inappropriate sampling. Moreover results of microbiological studies are not immediately available and in most cases the turnaround time exceeds the time line for a swift diagnosis. A biomarker like PCT that possesses rapid kinetics and has acceptable diagnostic accuracy for identifying sepsis is needed for laboratories as well as clinicians.

To the best of our knowledge this is the first study exploring the accuracy of PCT for the diagnosis of bacterial sepsis in critically ill adult patients in ICUs from Pakistan. Most studies from Pakistan aimed at diagnostic utility of PCT are undertaken in the neonates and children [5,16]. Our study population was a large, diverse group of critically ill adult patients with sepsis, admitted to the ICU. Patients were classified into having suspected sepsis on the basis of the 1991 ACCP/SCCM Sepsis Directory and the diagnostic criteria advanced by the 2001 International Sepsis Definition Conference [17]. Furthermore, these findings were also confirmed by the ICU consultants.

Our study reported a male predominance with sepsis (60%), another study conducted at our centre undertaken over a period of two years from 2012 to 2013 has reported that 209 patients admitted to the surgical ICU had sepsis; with a male predominance of 67.6% which is correlating with our findings [18]. Similarly studies from other centres and a multicentre trial from India also reported higher rates of sepsis associated with male gender compared to females [19,20].

Additionally, higher WBC counts reported in our study are in agreement with various studies which have also evaluated the efficacy of WBC as a marker of sepsis and have reported higher counts in case of culture proven sepsis [[21], [22], [23]].

Based on data collected by the European Antimicrobial Resistance Surveillance Network (EARS-Net) and the former EARSS, *Escherichia coli* and *Staphylococcus aureus* are the main causes of bloodstream infections in humans [24]. And our study seconds these findings.

The PCT cut off employed by ICUs worldwide for diagnosis of sepsis greatly varies. At a serum PCT cutoff of 0.5 μg/L, our study has reported a sensitivity and specificity of 93.75% and 43.59% respectively. The diagnostic efficacy of PCT was further tested with receiver operating characteristic curves compared to culture which revealed an AUC of 0.7. The cut off that maximizes the area under the curve for sepsis diagnosis is found to be 1.5 μg/L. This cut off has a better specificity compared to a cut off 0.5 μg/L. Furthermore, this is in concordance with various published studies. Results summarized in Table 3.

**Table 3:** Comparison of diagnostic accuracy of PCT for diagnosis of sepsis with other studies at a cut off of 0.5 μg/L and 1.5 μg/L.

Comparison of diagnostic accuracy of PCT for diagnosis of sepsis with other studies at a cut off of 0.5 μg/L				
	Country	n	Sensitivity [%]	Specificity [%]
Aw TC et al^.^ [25]	Singapore	626	79.6	47.2
Bossink et al. [26]	Netherlands	133	65	58
Luyt et al. [27]	France	172	72	24
Sudhir et al. [28]	India	100	94	Not reported
Alzahrani et al. [29]	Saudi Arabia	109	54.4	65.9
Our study	Pakistan	103	93.8	43.5
**Comparison of diagnostic accuracy of PCT for diagnosis of sepsis with other studies at a cut off of 1.5 μg/L**				
	**Country**	**n**	**Sensitivity** [**%]**	**Specificity [%]**
Du et al. [30]	China	51	80	74
Wanner et al. [31]	Switzerland	133	76	77
Tugrul et al. [32]	Turkey	85	73	80
Our study	Pakistan	103	64	70

Therefore, we recommend that for differentiating bacterial sepsis from other causes of sepsis a cut-off of 1.5 μg/L should be utilized by adult ICUs in our population.

There were some limitations of this study; serial measurement of PCT were not undertaken to report the kinetics and PCT could also be falsely negative in early course of infection which warrants treatment. Additionally any localized infections e.g. osteomyelitis, abscess can also present with PCT values below the cut off. Antibodies testing for viral infections were not done; similarly fungal cultures were not performed to rule out fungal infections. The sample size was small limiting the power of the present study and there is a likelihood that large scale studies in Pakistani ICU may differ from our findings. A small subset of patients (n = 12) in our study however presented with markedly elevated procalcitonin levels but negative culture results ranging from 3.12 to 100 μg/L. Most of the patients in this group presented with sepsis secondary to respiratory tract infections (RTIs) (n = 8). Blood cultures are reported to be less accurate predictors of RTIs compared to sputum culture and might lead to discrepant outcomes [33]. Another factor to consider in our study setup was the possibility of patients receiving antibiotic treatment before cultures were obtained, hence resulting in false-negative culture results. Furthermore one patient in our study with elevated PCT presented with malarial infection and negative cultures, which is in concordance with other studies that have witnessed elevation of PCT in malaria [34,35].

This study demonstrates PCT to be promising marker of sepsis in critically ill patients. However we recommend that PCT values must be considered in conjunction with clinical examination and other laboratory tests while implementing the therapeutic plan. Thus to conclude adding PCT to the list of standard laboratory work up of critically ill patients with suspected sepsis could increase diagnostic accuracy leading to improved patient care.

## Provenance and peer review

Not commissioned, externally peer reviewed.

## Ethical approval

Study was approved by the institutional Ethical Review Committee (1573-Pat-ERC-11) of the Aga Khan University.

## Sources of funding

The Departmental Resident Research Grant (I.D: RRG # 2013–06), Department of Pathology and Laboratory Medicine, the Aga Khan University.

## Author contribution

Dr Sibtain Ahmed performed the literature search, data collection and majority of the writing work in the first draft. Dr Aysha Habib Khan helped in designing the outline and reviewed the final draft. Dr Madiha Hashmi assisted in patient recruitment and contributed in writing the first draft. Dr Lena Jafri, Dr Imran Siddiqui helped in data analysis and contributed in writing and final review of the manuscript. Dr Farooq Ghani conceived the idea and coordinated the writing of the paper. All authors have reviewed the final draft and agreed upon.

## Conflicts of interest

There is no conflict of interest associated with any of the authors. We have had no involvements that might raise the question of bias in the work reported or in the conclusions.

## Research registration number

NCT03506152.

## Guarantor

Dr Aysha Habib Khan.

Associate Professor & Consultant Chemical Pathologist.

Department of Pathology & Laboratory Medicine.

Aga Khan University.

Stadium Road.

Karachi, Pakistan.

## References

[1] Magrini L., Gagliano G., Travaglino F., Vetrone F., Marino R., Cardelli P. (2014 Oct). Comparison between white blood cell count, procalcitonin and C reactive protein as diagnostic and prognostic biomarkers of infection or sepsis in patients presenting to emergency department. Clin. Chem. Lab. Med..

[2] Vincent J.L., Marshall J.C., Namendys-Silva S.A., Francois B., Martin-Loeches I., Lipman J. (2014 May). Assessment of the worldwide burden of critical illness: the intensive care over nations (ICON) audit. Lancet Respir Med.

[3] Phua J., Koh Y., Du B., Tang Y.Q., Divatia J.V., Tan C.C. (2011 Jun 13). Management of severe sepsis in patients admitted to Asian intensive care units: prospective cohort study. BMJ.

[4] Previsdomini M., Gini M., Cerutti B., Dolina M., Perren A. (2012). Predictors of positive blood cultures in critically ill patients: a retrospective evaluation. Croat. Med. J..

[5] Khan D.A., Rahman A., Khan F.A. (2010). Is procalcitonin better than C-reactive protein for early diagnosis of bacterial pneumonia in children?. J. Clin. Lab. Anal..

[6] Yap C.Y.T.A. (2014). The use of procalcitonin in clinical practice. Proceedings of Singapore Healthcare.

[7] Karlsson S., Heikkinen M., PettilÃ¤ V., Alila S., VÃ¤isÃ¤nen S., Pulkki K. (2010). Predictive value of procalcitonin decrease in patients with severe sepsis: a prospective observational study. Crit. Care.

[8] Ruiz-Alvarez M.J., GarcÃ-a-Valdecasas S., De Pablo R., GarcÃ-a M.S., Coca C., Groeneveld T.W. (2009). Diagnostic efficacy and prognostic value of serum procalcitonin concentration in patients with suspected sepsis. J. Intensive Care Med..

[9] Assicot M., Bohuon C., Gendrel D., Raymond J., Carsin H., Guilbaud J. (1993). High serum procalcitonin concentrations in patients with sepsis and infection. Lancet.

[10] Kim J.H., Seo J.W., Mok J.H., Kim M.H., Cho W.H., Lee K. (2013). Usefulness of plasma procalcitonin to predict severity in elderly patients with community-acquired pneumonia. Tuberc. Respir. Dis..

[11] Tamminga C.A., Nemeroff C.B., Blakely R.D., Brady L., Carter C.S., Davis K.L. (2002). Developing novel treatments for mood disorders: accelerating discovery. Biol. Psychiatry.

[12] Jeong S., Park Y., Cho Y., Kim H.-S. (2012). Diagnostic utilities of procalcitonin and C-reactive protein for the prediction of bacteremia determined by blood culture. Clin. Chim. Acta.

[13] Agha R.A., Borrelli M.R., Vella-Baldacchino M., Thavayogan R., Orgill D.P. (2017 Oct). The STROCSS statement: strengthening the reporting of cohort studies in Surgery. Int. J. Surg..

[14] Vincent J.L., Sakr Y., Sprung C.L., Ranieri V.M., Reinhart K., Gerlach H. (2006 Feb). Sepsis in European intensive care units: results of the SOAP study. Crit. Care Med..

[15] Lai P.P., Leung A.K., Li A.N., Zhang M. (2008). Three-dimensional gait analysis of obese adults. Clin. Biomech..

[16] Dilshad Ahmad Khan A.R., Khan Farooq Ahmed, Najm-ul-Hassan (2009 June 2009). Comparison of serum Procalcitonin and C-reactive protein in diagnosis of Bacterial meningitis. Pakistan Armed Forces medical Journal. [original].

[17] Bone R.C., Balk R.A., Cerra F.B., Dellinger R.P., Fein A.M., Knaus W.A. (1992). Definitions for sepsis and organ failure and guidelines for the use of innovative therapies in sepsis. The ACCP/SCCM consensus conference committee. American College of chest physicians/society of critical care medicine. Chest Journal.

[18] Asghar A., Hashmi M., Rashid S., Khan F.H. (2016 Jan-Mar). Incidence, outcome and risk factors for sepsis--a two year retrospective study at surgical intensive care unit of a teaching hospital in Pakistan. J. Ayub Med. Coll. Abbottabad.

[19] Mayr F.B., Yende S., Angus D.C. (2014). Epidemiology of severe sepsis. Virulence.

[20] Todi S. (2010). Sepsis: new horizons. Indian J. Crit. Care Med.: peer-reviewed.

[21] Zhang D., Micek S.T., Kollef M.H. (2015). Time to appropriate antibiotic therapy is an independent determinant of postinfection ICU and hospital lengths of stay in patients with sepsis*. Crit. Care Med..

[22] Linder A., Akesson P., Inghammar M., Treutiger C.-J., LinnÃ©r A., SundÃ©n-Cullberg J. (2012). Elevated plasma levels of heparin-binding protein in intensive care unit patients with severe sepsis and septic shock. Crit. Care.

[23] Nierhaus A., Klatte S., Linssen J., Eismann N.M., Wichmann D., Hedke (2013). Revisiting the white blood cell count: immature granulocytes count as a diagnostic marker to discriminate between SIRS and sepsis-a prospective, observational study. BMC Immunol..

[24] Gagliotti C., Balode A., Baquero F., Degener J., Grundmann H., Gur D. (2011). Escherichia coli and Staphylococcus aureus: Bad News and Good News from the European Antimicrobial Resistance Surveillance Network.

[25] Aw T.C.Y.C., Yew L.S., Lim W.R., Leow P.Q., Tan S.R. (2009 01 June). The use of a new rapid procalcitonin for the evaluation of patients with infection. Clinical Chemistry. [absrtact].

[26] Bossink A.W.J., Groeneveld A.B.J., Thijs L.G. (1999). Prediction of microbial infection and mortality in medical patients with fever: plasma procalcitonin, neutrophilic elastase-Î±1-antitrypsin, and lactoferrin compared with clinical variables. Clin. Infect. Dis..

[27] Luyt C.-E., Combes A., Reynaud C., Hekimian G., Nieszkowska A., Tonnellier M. (2008). Usefulness of procalcitonin for the diagnosis of ventilator-associated pneumonia. Intensive Care Med..

[28] Sudhir U., Venkatachalaiah R.K., Kumar T.A., Rao M.Y., Kempegowda P. (2011). Significance of serum procalcitonin in sepsis. Indian J. Crit. Care Med.: peer-reviewed.

[29] Alzahrani A.J. (2009). Rapid detection of procalcitonin as an early marker of sepsis in intensive care unit in a tertiary hospital. Int. J. Med. Med. Sci..

[30] Du B., Pan J., Chen D., Li Y. (2003). Serum procalcitonin and interleukin-6 levels may help to differentiate systemic inflammatory response of infectious and non-infectious origin. Chinese Med J.

[31] Wanner G.A., Keel M., Steckholzer U., Beier W., Stocker R., Ertel W. (2000). Relationship between procalcitonin plasma levels and severity of injury, sepsis, organ failure, and mortality in injured patients. Crit. Care Med..

[32] Tugrul S., Esen F., Celebi S., Ozcan P.E. (2002). Reliability of procalcitonin as a severity marker in critically ill patients with inflammatory response. Anaesth. Intensive Care.

[33] Zhu Y., Yuan Y., Huang H. (2015). Comparison of serum procalcitonin in respiratory infections and bloodstream infections. Int. J. Clin. Exp. Med..

[34] Manegold C., Schmiedel S., Chiwakata C.B., Dietrich M. (2003). Procalcitonin serum levels in tertian malaria. Malar. J..

[35] Hesselink D.A., Burgerhart J.-S., Bosmans-Timmerarends H., Petit P., van Genderen P.J. (2009). Procalcitonin as a biomarker for severe Plasmodium falciparum disease: a critical appraisal of a semi-quantitative point-of-care test in a cohort of travellers with imported malaria. Malar. J..

